# The interactions between oral-gut axis microbiota and *Helicobacter pylori*

**DOI:** 10.3389/fcimb.2022.914418

**Published:** 2022-08-03

**Authors:** Xi Chen, Nanxi Wang, Jiannan Wang, Binyou Liao, Lei Cheng, Biao Ren

**Affiliations:** ^1^ State Key Laboratory of Oral Diseases & National Clinical Research Center for Oral Diseases & West China School of Stomatology, Sichuan University, Chengdu, China; ^2^ Department of Operative Dentistry and Endodontics, West China Hospital of Stomatology, Sichuan University, Chengdu, China

**Keywords:** *Helicobacter pylori*, oral-gut axis, oral microbiota, gut microbiota, interactions between microorganisms

## Abstract

In the human body, each microbial habitat exhibits a different microbial population pattern, and these distinctive microflorae are highly related to the development of diseases. The microbial interactions from host different niches are becoming crucial regulators to shape the microbiota and their physiological or pathological functions. The oral cavity and gut are the most complex and interdependent microbial habitats. *Helicobacter pylori* is one of the most important pathogens from digestive tract, especially the stomach, due to its direct relationships with many gastric diseases including gastric cancer. *H. pylori* infections can destroy the normal gastric environment and make the stomach a livable channel to enhance the microbial interactions between oral cavity and gut, thus reshaping the oral and gut microbiomes. *H. pylori* can be also detected in the oral and gut, while the interaction between the oral-gut axis microbiota and *H. pylori* plays a major role in *H. pylori*’s colonization, infection, and pathogenicity. Both the infection and eradication of *H. pylori* and its interaction with oral-gut axis microbiota can alter the balance of the microecology of the oral-gut axis, which can affect the occurrence and progress of related diseases. The shift of oral-gut axis microbiota and their interactions with *H. pylori* maybe potential targets for *H. pylori* infectious diagnosis and treatment.

## Introduction

The balance and dysbiosis of the human microbiome are inextricably associated with health and disease (Hou et al., 2021). There are many different and specific microbial habitats in the human body. Each microbial habitat shows a different microbial population pattern, and the microbial interactions within the same niche or from different niches are important for the microecological balance and host health (Baquero et al., 2021). The oral cavity and gut are the most complex microbial habitats. The interaction between oral and gut microbiota is complicated, unstable, and interconnected (Acharya et al., 2017). Under normal physiological conditions, they can maintain a fine-tuned balance, but the imbalance of crosstalk will contribute to the occurrence and development of diseases (Albuquerque-Souza and Sahingur, 2022).

The transmission of oral to gut and gut to oral microorganisms can shape and/or reshape the microbial ecosystem in both habitats and thus regulate the pathogenesis of different diseases (Park et al., 2021), especially in cases of oral-gut barrier damage (Khor et al., 2021). The composition of the gut microbiota was similar to the oral microbiome under low gastric acidity condition caused by the long-term usage of proton pump inhibitors and the urease produced by infected *Helicobacter pylori*, further suggesting the interorgan translocation of the oral and gut microbiota due to the oral-gut barrier dysfunction (Park et al., 2021). *H. pylori*, a gram-negative human pathogen, has infected approximately fifty percent of humans worldwide (Lee et al., 2022). It is one of the most studied bacteria which can survive stably in the gastric acid environment and has co-evolved with humans for thousands of years (Kang and Blaser, 2006). Due to its strong correlation with gastric cancer, the World Health Organization’s International Agency for Research on Cancer (IARC) classified *H. pylori* as “Group 1” carcinogen to humans. *H. pylori* infection was capable to change the pH of the gastric environment (Camilo et al., 2017), and it can transmit through the oral-oral and fecal-oral route (Mégraud, 1995) to cause gastric diseases including chronic gastritis, gastric ulcer, gastric adenocarcinoma, etc. (Tsay and Hsu, 2018). The abundance of *H. pylori* in the mouth is very low compared to that in the stomach as it constituted approximately 42% – 97% of the total gastric bacterial community (Schulz et al., 2018), but *H. pylori* can significantly affect the oral community while some other microorganisms can also affect its colonization in the oral cavity (Vasapolli et al., 2019). *H. pylori* affects the microbiota and diseases of the oral-gut axis (Mladenova and Durazzo, 2018) as it connects the entire gastrointestinal tract through its transmission route from mouth to the stomach.

## *Helicobacter pylori* and oral microbiome

### Oral microbiome composition

As the initiation point of digestion, the oral cavity with its unique niches, such as the gingival sulcus, the tongue, the hard and soft palates, the saliva, and the teeth, is an exceptionally complex habitat that presents over 700 species of microorganisms including bacteria, fungi, viruses, and protozoa (Mark Welch et al., 2019). Actinobacteria, Bacteroidetes, Firmicutes, Proteobacteria, TM7 (Saccharibacteria), and Spirochaetes are common bacteria at the phylum level, while *Fusobacterium*, *Gemella*, *Haemophilus*, *Neisseria*, *Porphyromonas*, *Prevotella*, *Streptococcus*, *Veillonella*, *Actinomyces*, *Alloprevotella*, *Pseudomonas*, *Treponema*, *Solobacterium* are common at the genus level, which can be found in all the oral sites of healthy subjects (Sharma et al., 2018b; Park et al., 2021). In particular, *Streptococcus* was the most abundant genus which represented 12% to 66% of the total genera detected in the oral cavity. The abundance of *Neisseria*, *Prevotella*, and *Haemophilus* genera were also high, counting for 6% to 29% of the total bacteria detected (Caselli et al., 2020). The oral microbiome is maintained in homeostasis in the healthy state of the host (Lamont et al., 2018), but the occurrence of host diseases will lead to the imbalance of oral flora, indicating that the oral microbiome can directly reflect the host health conditions (Hakansson et al., 2018).

### Effects of oral microbiome on *Helicobacter pylori*

**Figure 1:**
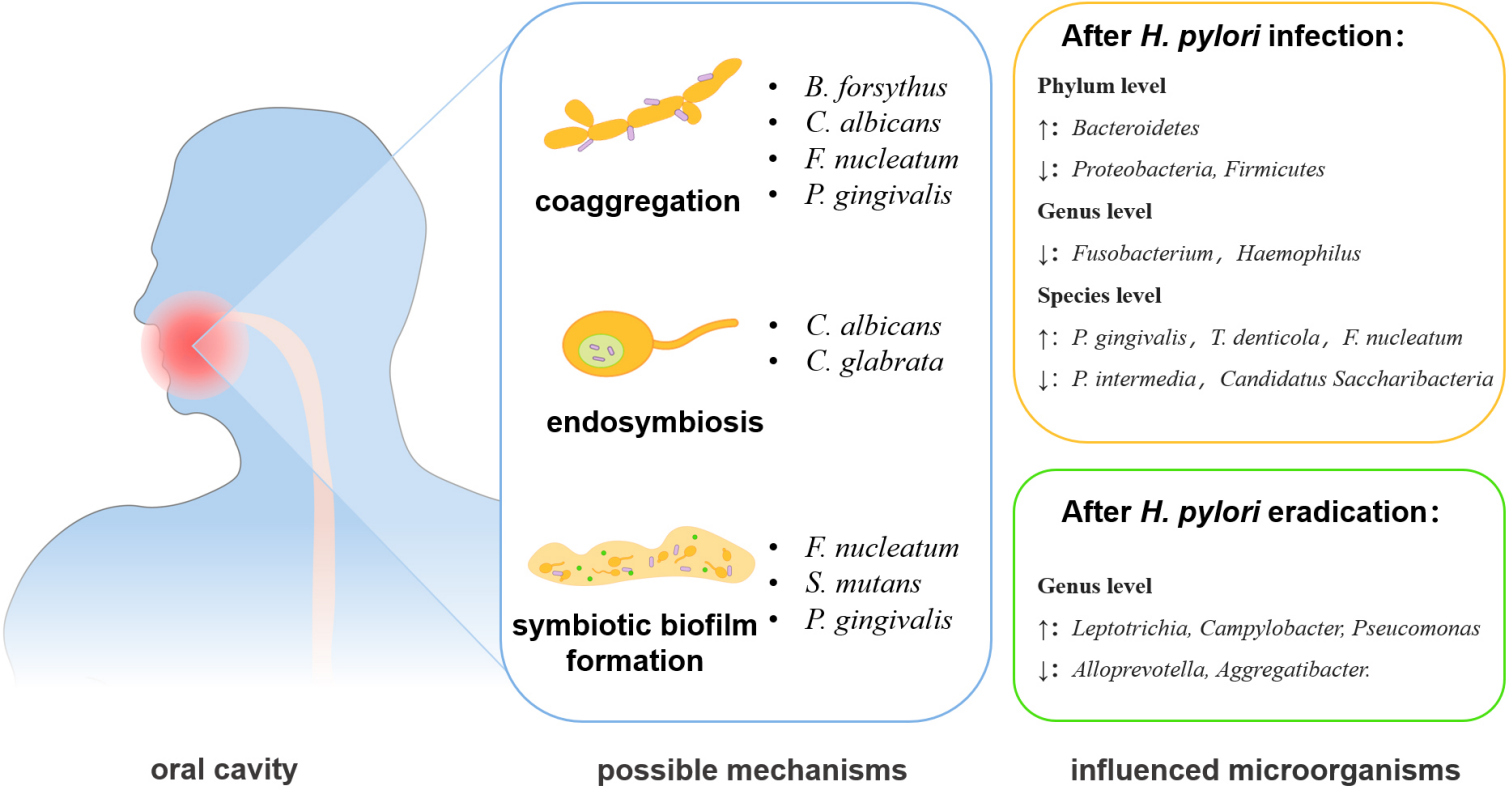
The changes and interactional mechanisms of *H. pylori* and oral microbiota. The interactions between *H. pylori* and oral microbiome may act through co-aggregation, endosymbiosis, and formation of symbiotic biofilm. The eradication of *H. pylori* infection can also affect the oral microbiota.

The oral and gastric environments are linked together by saliva and digested food (Freitas et al., 2018). A recent study indicated that oral microbiome was the main source of gastric microbes and was closely related to the infection and transmission of *H. pylori* (Wu et al., 2021) (**Table 1**), and oral microorganisms can impact the transmission and colonization of *H. pylori* (Kivi and Tindberg, 2006). The main interaction patterns are coaggregation, symbiotic biofilm formation, endosymbiosis, etc. (Nobbs and Jenkinson, 2015; Chen et al., 2021b) (**Figure 1**). *Fusobacterium nucleatum* and *Porphyromonas gingivalis*, key bacteria in periodontal diseases, can aggregate with *H. pylori* cells and the coaggregation was inhibited by EDTA, lysine, or arginine *in vitro*, indicating the potential promotion of *H. pylori* oral-to-stomach colonization by oral bacteria (Okuda et al., 2003; Park et al., 2016). *Streptococcus mutans*, the major cariogenic bacterium, can form a symbiotic biofilm with *H. pylori* to increase its survival in the unsuitable environment of the mouth (Nomura et al., 2020).

**Table 1:** Studies assessing the influence of *Helicobacter pylori (H. pylori*) infection on oral microbiota.

Author, year	Study groups	Age	Sample	Microorganisms’ changes after *H. pylori* infection		Main findings
				Increased	Decreased	
(Li et al., 2021)	Oral lichen planus (OLP) and negative control (NC): 21 were *H. pylori* (+) OLP, 9 were *H. pylori* (−) OLP, 11 were *H. pylori* (+) NC, 10 were *H. pylori* (−) NC	Adult	Saliva	phylum: Bacteroidetes genus: *Alloprevotella*, *Haemophilus*	phylum: Proteobacteria, Firmicutes genus: *Actinomyces*	• *H. pylori* affects erosive OLP by inducing the secretion of cytokines IL-6, IL-17, and IL-8, which causes the abundance of oral microorganisms in OLP patients to change.
(Ji et al., 2020)	34 were *H. pylori* (+), 24 were *H. pylori* (−)	Adult	Saliva	*Acinetobacter*, *Ralstonia*, *Leptothrix*, *Sphingomonas*, *Ochrobactrum*, *Afipia*, *Leptotrichia*, *Oribacterium*, *Moryella*	*Alloprevotella*, *Aggregatibacter*, *Klebsiella*, *Leptotrichlaceae:G_1_*, *Fusobacterium*, *Parvimonas*, *Peptococcus*	• *H. pylori* produces large amounts of urease, which reduces the acidic environment in the stomach thereby altering the oral microbial community and structure.
(Kadota et al., 2020)	29 were *H. pylori* (+), 10 were *H. pylori* (−)	Adult, elder	Saliva, dental plaque, dental pulp	*P. gingivalis*, *T. denticola*, *T. forsythia*	*P. intermedia*, *Prevotella nigrescens*, *Campylobacter rectus*	• The planting of *H. pylori* in the oral cavity related to the existence of the red complex (*P. gingivalis*, *T. denticola*, and *T. forsythia*). • Low pathogenic periodontal bacteria have an inhibitory effect on *H. pylori*, such as orange complex (*P. intermedia*, *P. nigrescens*, and *C. rectus*), and green complex (*Capnocytophage ochracea*, *Capnocytophage sputigena*, *A. actinomycetemcomitans*, and *Eikenella corrodens*). • *H. pylori* is associated with the formation of periodontal pockets.
(Zhao et al., 2019)	Gastritis: 13 were (CagA−) *H. pylori* (+), 35 were (CagA +) *H. pylori* (+), 32 were *H. pylori* (−)	Adult, elder	Tongue plaque	After (CagA−) *H. pylori* infection		• CagA positive strains of *H. pylori* can reduce the structural complexity of oral microorganisms, leading to a reduction in structural stability.
				Bacteroidetes, Firmicutes, Fusobacteria	Actinobacteria, Proteobacteria	
				After (CagA +) *H. pylori* infection		
				Actinobacteria, Proteobacteria	Bacteroidetes, Firmicutes, Fusobacteria	
(Chua et al., 2019)	10 were *H. pylori* (+), 14 were *H. pylori* (−)	Adult	Cheek mucosa	*Pseudomonas*, *Roseomonas*	*Fusobacterium*, *Solobacterium*, *Streptococcus*, *Haemophilus*	• *H. pylori*-positive individuals show more differences than negative in both alpha and beta diversity during the daytime. • *H. pylori* disrupts the balance of the oral microbiota only during the day by affecting systemic metabolic and immune factors. • *H. pylori* secretes proteins and metabolites, as well as alters the nutrient supply and pH in the oral cavity through proliferation, which affects the growth and structure of the oral microbiome during the day.
(Schulz et al., 2018)	Gastritis: 16 were *H. pylori* (+), 24 were *H. pylori* (−)	Adult, elder	Saliva	genus: *Treponema*	genus: *Haemophilus* species: *P. acnes*, *P. oris*	• Bacteria can migrate continuously through the upper gastrointestinal tract, demonstrating that saliva is a major source of gastric microorganisms.
(Hu et al., 2016)	Chronic periodontitis: 13 were *H. pylori* (+), 15 were *H. pylori* (−)	Adult	Plaque	*P. gingivalis*, *P. intermedia*, *F. nucleatum*, *T. denticola*	*A. actinomycetemcomitans*	• *H. pylori* infection increases the risk of periodontal disease by increasing the proportion of total periodontal pathogens in dental plaque.
(Umeda et al., 2003)	Once or now suffering from gastritis and peptic ulcer: 45 were *H. pylori* (+), 12 were *H. pylori* (−)	Adult, elder	Saliva, supragingival plaque, tongue plaque	*Bacteroides forsythus*, *A. actinomycetemcomitans*	*P. gingivalis*, *P. intermedia*	• Supragingival plaque and shallow periodontal pockets provide a good environment for *H. pylori* and also promote the co-aggregation of *H. pylori* with oral microorganisms, thus increasing the prevalence of *H. pylori*.
(Ishihara et al., 1997)	Peptic ulcer or gastritis: 54 were *H. pylori* (+), 48 were *H. pylori* (−)	Adult, elder	Saliva, plaque,	*F. nucleatum*, *P. gingivalis*	–	• Antagonism of oral bacteria against *H. pylori* can lead to its low detection rate
(Ji et al., 2020)	34 were *H. pylori* (+), 24 were *H. pylori* (−)	Adult	Saliva	**After *H. pylori* eradication**		• *H. pylori* produces large amounts of urease, which reduces the acidic environment in the stomach thereby altering the oral microbial community and structure.
				Increased	Decreased	
				phylum: Fusobacteria genus: *Leptotrichia*, *Campylobacter*, *Pseucomonas*	genus: *Alloprevotella*, *Aggregatibacter*	

*Candida albicans* is the most common fungus in the human body and its main habitats are the oral cavity, upper respiratory tract, and intestinal tract (D'Enfert et al., 2020). *C. albicans* can synergize with *H. pylori* to enhance its survival in an unfavorable living environment and promote its colonization and the infection (Chen et al., 2021b). *H. pylori* was found to enter *C. albicans* yeast cells in the oral cavity and vagina, while the intracellular *H. pylori* showed active motility even under high temperature, dryness, and antibiotics conditions (Saniee et al., 2013), indicating that the internalization synergistic relationship can protect *H. pylori* from unsuitable conditions. *H. pylori* can also anchor on the surface of the *C. albicans* and aggregate with *C. albicans* to form a mixed biofilm (Palencia et al., 2022).

Besides the synergistic interaction between *H. pylori* and oral microorganisms, some oral bacterial strains showed a hostile relationship with *H. pylori*, such as *S. mutans* JP2 and Ingbritt, *Streptococcus sobrinus* 6715, and three *Prevotella* species significantly inhibited the growth of *H. pylori in vitro*, and this growth inhibitory activity was affected by heat and protease treatments (Ishihara et al., 1997).

### Effect of *Helicobacter pylori* on the oral microbiome

*H. pylori* infection can disrupt oral microbiome homeostasis through the interplay with multiple members of the oral microbial community, such as *H. pylori* supernatant could inhibit *S. mutans* and *Streptococcus sanguinis* dual-species biofilm formation and their EPS production *in vitro* studies, but enhance the acid production of *S. mutans* to increase the abundance of *S. mutans* in this acidic condition as *S. mutans* is more acid resistant than *S. sanguinis* (Zhang et al., 2018). However, *H. pylori*-induced oral microbiome changes may differ under different oral samples and various host health conditions. Without oral and gastrointestinal diseases, several studies have determined the different changes of the microflora caused by *H. pylori* from different oral ecological niches. By sequencing the bacterial 16S rRNA gene V3-V4 hypervariable regions in saliva samples, the alpha diversity of *H. pylori*-infected subjects was similar to that of uninfected subjects, but for buccal swab sample*s*, α and β diversity changed significantly in *H. pylori*-positive individuals compared to *H. pylori*-negative individuals (Ji et al., 2020). *H. pylori* infection had a significant effect on the abundance of both *Pseudomonas* and *Rosemonas* genera and significantly decreased the abundance of *Haemophilus*, and *Streptococcus* in cheek mucosa samples, but the saliva samples showed no significant changes (Chua et al., 2019). These results indicate that *H. pylori* infection showed different effects in oral niches.

The interaction between *H. pylori* and oral microorganisms can differ from that of asymptomatic *H. pylori*-positive people with oral disease or gastrointestinal disease. The increase of certain oral bacteria was positively correlated with the colonization of *H. pylori*. For example, the Bacteroidetes at phylum level increased in Oral lichen planus (OLP) patients with *H. pylori* infection, while there was a positive correlation between *H. pylori* infection and the relative abundance of *Haemophilus* and *Alloprevotella* at the genus level (Li et al., 2021). When *H. pylori* infection was accompanied by gastrointestinal diseases, such as gastritis, an increase in *Treponema* at the genus level was detected in the oral cavity (Schulz et al., 2018), but some oral microorganisms showed opposite changes in abundance, such as the decreasing trend of *Haemophilus* at the genus level, *Propionibacterium acnes*, *Prevotella oris*, *P. gingivalis*, and *Prevotella intermedia* at the species level were also observed in gastrointestinal disorders patients with *H. pylori* infection, indicating the different interaction between *H. pylori* and gastrointestinal diseases on oral microbiome (Umeda et al., 2003; Schulz et al., 2018).

The expression of virulence factors of *H. pylori* may also affect the oral microbiome. The abundance of Actinobacteria and Proteobacteria was increased, while the abundance of Bacteroidetes, Firmicutes, and Fusobacteria was decreased from the tongue plaque samples of CagA-positive *H. pylori-*infected patients, but these changes were totally reversed in the CagA-negative *H. pylori-*infected patients (Zhao et al., 2019).

The *H. pylori* eradication can also cause the changes of oral microbiome. In saliva samples from patients without severe oral diseases, such as periodontitis and OLP, the eradication treatment of *H. pylori* decreased the salivary bacterial diversity, but the genera *Lautropia*, Burkholderiales, Burkholderiaceae, and *Actinomyces* were enriched (Ji et al., 2022). Another study found that the eradication of *H. pylori* was followed by a relative increase of most oral bacteria, including *Ralstonia*, *Leptotrichia*, *Sphingomonas*, *Leptothrix*, *Oribacterium*, and *Acinetobacter*, except for *Ochrobactrum* (Ji et al., 2020). More importantly, the experiment also revealed that *H. pylori* eradication exacerbates the changes in oral microorganisms caused by *H. pylori* infection, for example, *Alloprevotella*, *Aggregatibacter*, *Leptotrichlaceae:G_1_*, *Parvimonas*, and *Fusobacterium* would further decrease in number (Ji et al., 2020). In conclusion, the infection/eradication of *H. pylori* can change the structure of oral microorganisms, and thus may affect the development and progress of oral diseases.

### Oral diseases and *Helicobacter pylori*

The infection of *H. pylori* is highly correlated with oral diseases (Perez-Perez et al., 2004; Anand et al., 2006), and the shift of oral microbial community induced by *H. pylori* is one of the potent reasons for the oral diseases (Martin and Solnick, 2014). Periodontitis, a common microbiome-driven inflammatory disease (Hajishengallis, 2022), was highly related to *H. pylori* infection. *P. gingivalis*, as an established pathogenic agent of periodontitis (Miller and Scott, 2021), had a positive correlation with *H. pylori*. In chronic periodontitis patients with *H. pylori* infection, the red complex associated with periodontal disease was significantly increased in plaque, including *P. gingivalis, Treponema denticola, Tannerella forsythia* (Hu et al., 2016), indicating that *H. pylori* infection may promote periodontal disease. However, the orange and green complex showed low abundance in *H. pylori*-positive individuals (Kadota et al., 2020). *H. pylori* and increased *P. gingivalis* due to *H. pylori* infection, can both produce heat shock protein 60 (HSP60), which can target human HSP60 and aggravate the progression of periodontitis (Matsuura et al., 2008; Rizzo et al., 2012). *H. pylori* infection was also closely correlated to the erosive OLP (Li et al., 2021) as *H. pylori* infection increased the production of inflammatory cytokines IL-6 and IL-8, while these inflammatory cytokines may regulate the oral immune microenvironment through blood to exacerbate the inflammatory response in oral cavity (Du Teil Espina et al., 2019). There was also a strong correlation between *H. pylori* infection and halitosis, but the mechanism was not clear as it was difficult to determine whether the halitosis was induced by *H. pylori* in the stomach or caused by the changes of oral microflora induced by *H. pylori* infection (Anbari et al., 2019). A study analyzed the correlation between Behçet’s syndrome (BS) and *H. pylori* infection and found that BS patients had a higher rate of *H. pylori* infection, and the clinical symptoms including oral ulceration, genital ulceration, and cutaneous lesions could be improved after *H. pylori* eradication (Yu et al., 2019). However, its specific mechanism remains to be explored.

*H. pylori* infection is closely related to a variety of oral diseases, but traditional antibiotic therapy is increasingly difficult to eradicate *H. pylori* in the stomach, however, periodontal therapy, adjunctive treatment of traditional antibiotic therapy, had been shown to play an important role in the eradication of gastric *H. pylori* (Ren et al., 2016). It can also effectively reduce the oral pathogenic bacteria enriched by *H. pylori* infection to achieve the prevention and treatment of oral diseases (Okuda et al., 2003).

## *Helicobacter pylori* and gut microbiome

### Gut microbiome

The gut is the largest microbial ecosystem in the human body which contains approximately 500 to 1000 species in more than 50 different phyla (Qin et al., 2010). The human gut microbiome is established early in life and can be altered by host diet, lifestyle factors, and health status (Shanahan et al., 2021). The childhood/adolescent gut communities are enriched in *Bifidobacterium* spp., *Faecalibacterium* spp., and members of the Lachnospiraceae family (Hollister et al., 2015). In contrast, the adult gut microbiome is more stable, and it is seemed that the environmental factors play a much greater role than genetic factors (Spor et al., 2011). The gut microbiota, mainly anaerobic, consists of five major phyla including Actinobacteria, Bacteroidetes, Firmicutes, Proteobacteria, and Verrucomicrobia, while Cyanobacteria and Fusobacteria are presented in a minor proportion (Sekirov et al., 2010; Ghosh and Pramanik, 2021). Various disease states are directly related to the gut microbial diversity and their functions (Baruch et al., 2021; Davar et al., 2021), therefore, gut microbiota can reflect host age, health conditions, behaviors, and lifestyles (Zmora et al., 2019).

### Effect of *Helicobacter pylori* on the gut microbiome

**Figure 2:**
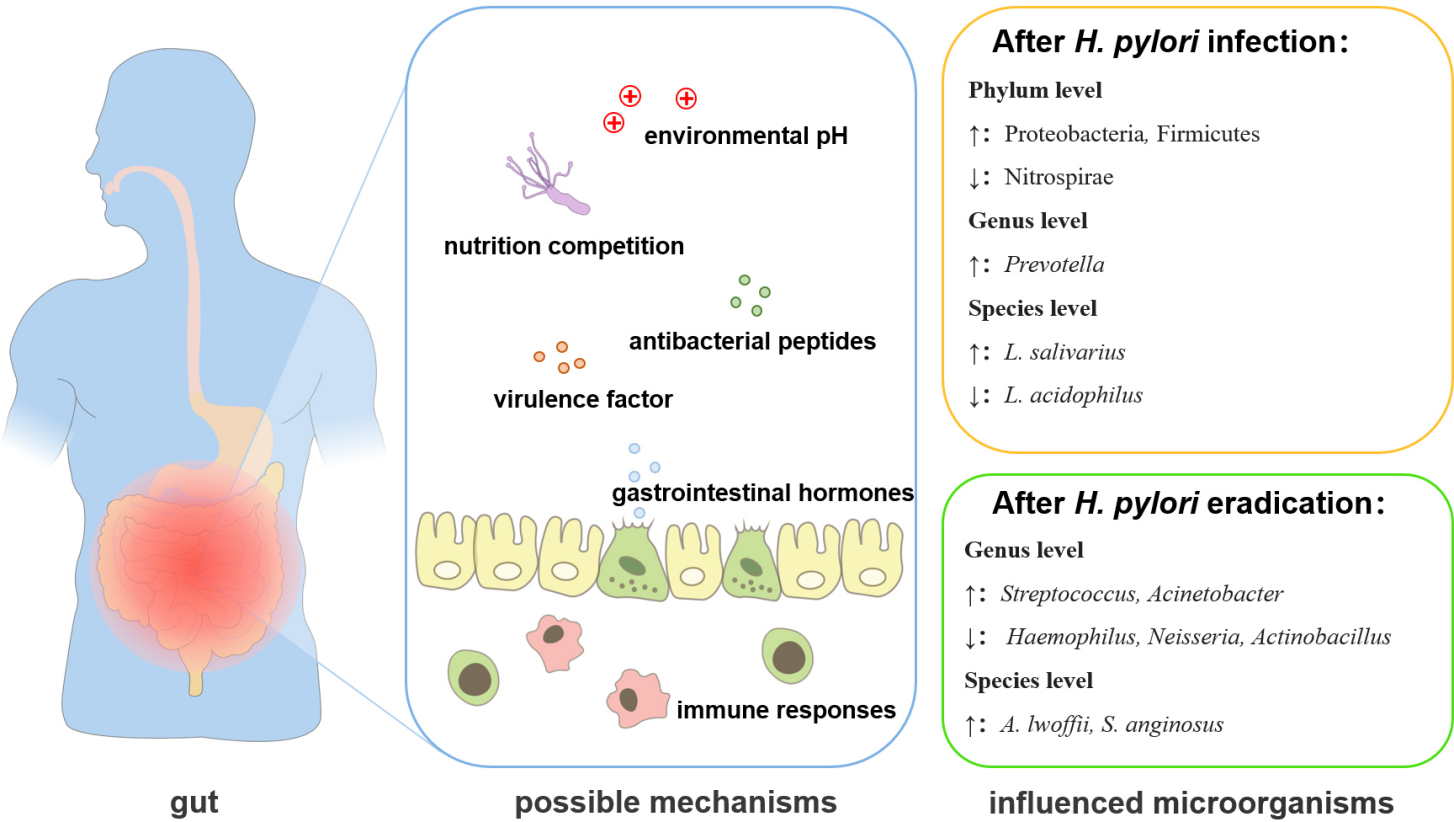
The changes and interactional mechanisms of *H. pylori* and gut microbiota. *H. pylori* infection can regulate the gut microbiota through 1. secretion of virulence factors; 2. mobilizing antibacterial peptides; 3. nutrition competition; 4. inducing or reducing the secretion of gastrointestinal hormones; 5. changing the pH of the environment; 6. affecting the immune response.7. eradication of infection.

As an important member of the gut ecosystem, *H. pylori* can influence the gastrointestinal microbiota through host-microbial or microbial-microbial interactions (Schulz et al., 2018; Noto et al., 2019) (**Table 2**). *H. pylori* can regulate the gut microenvironment in different ways (**Figure 2**). The virulence factors secreted by *H. pylori* may affect the gut microbiota. In a transgenic Drosophila model heterologously expressed the *H. pylori* virulence factor CagA, the expression of CagA alone shifted the gut microbial community, such as the proliferation of *Lactobacillus brevis* was significantly increased (Jones et al., 2017). *H. pylori* infection could also induce the secretion of different gastrointestinal hormones (He et al., 2016). The increased gastrin release was found in *H. pylori*-positive subjects and the hormone levels remodeled the intestinal metabolism, thereby affecting the gut microbiome, such as the level of leptin was positively correlated with the quantity of *Bifidobacterium* and *Lactobacillus* (Mohammadi et al., 2020). *H. pylori* may also influence the abundance of *Lactobacillus, Allobaculum*, *Turicibacter*, and *Anaeroplasma* by elevating the secretion of ghrelin (Kienesberger et al., 2016). *H. pylori* can affect the gut microbiota by changing the pH of the colonic environment. *H. pylori* decreased the acidity of the colon to cause a decrease in Bacteroidetes and an increase in Firmicutes and Proteobacteria in the fecal microbiota (Gao et al., 2018; Hold and Hansen, 2019). The infection o*f H. pylori* was accompanied by an increase of *Lactobacillus salivarius* and a decrease of *Lactobacillus acidophilus*, which was also related to the decrease of gastric acid secretion (Iino et al., 2018). *H. pylori* infection may also modulate the immune response to affect the gut microbiota (Ge et al., 2018). *H. pylori*-positive patients exhibited a decreased number of short-chain fatty acids (SCFAs) producing gut bacteria, which played important roles in modulating intestinal homeostasis (Smith et al., 2013), leading to the enrichment of *Prevotella copri* (Sitkin et al., 2022). In addition, *H. pylori* infection also affected the growth of many gut microorganisms, commonly including *Desulfovibrio*, *Prevotella*, *Haemophilus*, *Bacteroides*, *Parasutterella*, *Pseudoflavonifractor* at the genus level, *Candida glabrata*, *Enterobacter cloacae*, *Klebsiella pneumoniae*, *Sutterella wadsworthensis*, *Bacteroides vulgatus*, *Escherichia coli* at the species level (Dash et al., 2019; Frost et al., 2019; Martín-Núñez et al., 2019; Wang et al., 2019), but the mechanisms remain to be explored.

**Table 2:** Studies assessing the influence of *Helicobacter pylori (H. pylori*) infection on gut microbiota.

Author, year	Study groups	Age	Sample	Microorganisms’ changes				Main findings
				After *H. pylori* infection		After *H. pylori* eradication		
				Increased	Decreased	Increased	Decreased	
(Sung et al., 2020)	*H. pylori* (+) patients: 295 were BQT (+), 292 were BQT (−)	Adult, elder	Gastric biopsy tissue	–	–	genus: *Ralstonia*, *Granulicatella*, *Actinomyces*, *Rothia*, *Peptostreptococcus*, *Streptococcus*, *Abiotrophia*, *Parvimonas* species: *A. lwoffii*, *S. anginosus*	genus: *Haemophilus*, *Neisseria*, *Actinobacillus*	• *H. pylori* eradication promotes the enrichment of intestinal protective bacteria and facilitates the treatment of precancerous lesions.
(Wu et al., 2019)	duodenal ulcer (DU): 40 were *H. pylori* (+), 20 were *H. pylori* (−)	Adult, elder	Feces	genus: *Faecalibacterium*, *Ruminococcus*, *Escherichia*, *Akkermansia*	phylum: Gemmatimonadetes, Nitrospirae, Chlorobi, WS3 genus: *Bacteroides*, *Roseburia*, *Prevotella*, *Bifidobacterium*, *Actinobacteria*, *Caldithrix*, *Lachnospira*, *Termi*	–	–	• *H. pylori* eradication therapy significantly reduces gut microbial diversity of duodenal ulcers, which can be improved by supplementation with *Bacillus subtilis* and *Enterococcus faecium* (BSEF).
(Dash et al., 2019; Frost et al., 2019; Martín-Núñez et al., 2019; Wang et al., 2019)	392 were *H. pylori* (+), 465 were *H. pylori* (−)	Adult, elder	Feces	family: Coriobacteriaceae, Enterococcaceae, Rikenellaceae genus: *Succinivibrio*, *Turicibacter*, *Desulfovibrio*, *Prevotella*, *Haemophilus* species: *C. glabrata*, *P. copri*, *E. cloacae*, *K. pneumoniae*	genus: *Bacteroides*, *Parasutterella*, *Pseudoflavonifractor* species: *B*. *vulgatus*, *S*. *wadsworthensis*, *E*. *coli*	phylum: Bacteroidetes genus: *Megamonas* species: *Bacteroides fragilis*	phylum: Actinobacteria, Firmicutes, Proteobacteria family: Rikenellaceae, Streptococcaceae, Turicibacteraceae, Ruminococcaceae, Oxalobacteriaceae, Bifidobacteriaceae genus: *Butyricimonas*, *Streptococcus*, *Turicibacter*, *Oscillospira*, *Oxalobacter* species: *Eubacterium biforme*, *Oxalobacter formigenes*	• *H. pylori i*nfection indirectly causes vitamin B12 deficiency by affecting the categories and function of gut microorganisms. • Eradication of *H. pylori* affects bacteria associated with the regulation of glucose homeostasis in the gut microorganism and could be a new target for glycemic improvement. • The disorders of the gut microbial group caused by *H. pylori* infections can destroy the intestinal barrier and increase the susceptibility to the disease. • *H. pylori* infection increases the biodiversity of gut microorganisms, thus enhancing stability against external disturbances
(Iino et al., 2018; He et al., 2019)	gastritis: 236 were *H. pylori* (+), 531 were *H. pylori* (−)	Adult	Feces	species: *L. salivarius*	species: *L. acidophilus*	genus: *Lactobacillus*, *Prevotella*, *Streptococcus*, *Acinetobacter*, *Bacteroides*, *Bifidobacterium*, *Blautia*, *Lachnoclostridium*	genus: *Alistipes*	• After eradication of *H. pylori* by BQT, the abundance and diversity of gut microorganisms decreases in the short term, but gradually returns to the level of healthy individuals • *H. pylori* infection and subsequent atrophic gastritis reduces gastric acid secretion, resulting in compromised diversity and function of *Lactobacillus* in the gut microflora.
(Schulz et al., 2018)	gastritis:16 were *H. pylori* (+), 24 were *H. pylori* (−)	Adult, elder	Duodenal aspirate and biopsy tissue	phylum: Proteobacteria, Bacteroidetes	phylum: Firmicutes family: Rhodobacteriaceae, Lachnospiraceae genus: *Actinomyces* species: *Salmonella infantis*, *Campylobacter gracilis*, *Staphylococcus aureus*, *Enterococcus*	–	–	• *H. pylori* affects the bacterial community in the duodenum and distinguishes between host effects and sampling regions on the bacterial community.
(Gao et al., 2018)	24 were *H. pylori* current infection, 23 were *H. pylori* previous infection	Adult, elder	Feces	*Gemella*, *Erysipelotrichaceae_UCG_004*	*Acidovorax*, *Rhodococcus*	–	–	• Intestinal microbial homeostasis is affected by *H. pylori* infection, which leads to the promotion of gastrointestinal precancerous lesions.
(Chen et al., 2018)	35 were *H. pylori* (+) in 14-day BQT, 35 were *H. pylori* (+) in 14-day *Clostridium butyricum* supplemental BQT, 35 were *H. pylori* (−)	Adult, elder	Feces	–	Nitrospirae	Proteobacteria, Cyanobacteria	Firmicutes, Bacteroidetes, Verrucomicrobia, Lentispaerae	• Long-term fluctuations in the gut microbiome caused by the use of antibiotics to eradicate *H. pylori* are harmful to the organism, but probiotics can be supplemented to improve gastrointestinal symptoms.
(Benavides-Ward et al., 2018)	28 were *H. pylori* (+), 28 were *H. pylori* (−)	Children	Feces	phylum: Proteobacteria, Firmicutes genus: *Clostridium*, *Prevotella*	–	–	–	• *H. pylori-*positive children have a twofold chance of having an increased variety and number of microorganisms in their colon tract compared to normal individuals. • *H. pylori* infects the absorption of nutrients by gut microorganisms and increases susceptibility to intestinal diseases.

*H. pylori* eradication also affects gut bacteria through different mechanisms. For example, *H. pylori* may compete for the nutrition to affect the gut microbiota, as the capacities of nutrient metabolism were restored after the eradication of *H. pylori*, and the abundance of Actinobacteria, Bacteroidetes, Firmicutes, and Fusobacteria at the phylum level, *Lactobacillus*, and *Bifidobacterium* at the genus level were increased significantly (Tao et al., 2020). After the eradication of *H. pylori*, the pH in the stomach significantly decreased, which led to the decrease in the Bacteroidetes-to-Firmicutes ratio and the enrichment of *Bifidobacterium*-related taxa in gastrointestinal microbiota (Guo et al., 2020). The eradication of *H. pylori* was usually achieved by bismuth-containing quadruple therapy (BQT) (Rimbara et al., 2011). However, *H. pylori* eradication with BQT also reshaped the structure of the gut microbiota, including changes in bacterial abundance at the genus level and species level (He et al., 2019; Martín-Núñez et al., 2019; Sung et al., 2020). After the BQT, most of the changed bacteria returned to normal levels, except for those belonging to Ruminococcaceae, Lachnospiraceae, and *Eubacterium*, as some beneficial bacteria belonged to Lachnospiraceae and Ruminococcaceae, and the major butyrate-producing bacteria were still kept in a decreased level (Chen et al., 2018). Some pathogenic bacteria such as *Acinetobacter baumannii* NIPH60, *Klebsiella* sp., and *Haemophilus* sp. were also increased After the BQT (Chen et al., 2018). It is also worth noting that probiotic supplementation in BQT for *H. pylori* eradication, reversed the long-term influence in the gut microbiome caused by the use of antibiotics and improved gastrointestinal symptoms, although no significant differences in eradication rates were observed (Chen et al., 2018). However, whether the changes in gut microbiology after *H. pylori* eradication are related to *H. pylori* itself or BQT is still unclear and needs to be further investigated.

### Gut diseases and *Helicobacter pylori*

The disturbance of the oral-gut axis microbiota is closely related to the occurrence and development of gut diseases, including inflammatory bowel disease (IBD) and colorectal cancer (CRC), etc. (Park et al., 2021), while *H. pylori* infection enriched some bacteria played key roles in gut diseases, such as *F. nucleatum*, *P. gingivalis*, and so on. IBD, including Crohn’s disease (CD) and ulcerative colitis (UC), is closely linked with oral-gut axis microbiome dysbiosis (Lee and Chang, 2021). IBD patients exhibited the increase of intestinal epithelial permeability due to the impacts on mucosal barrier (Sharma et al., 2018a). *F. nucleatum*, a common bacterium in oral cavity, which can be increased after *H. pylori* infection, was significantly enriched in the gut of IBD patients, but rarely in the healthy individuals (Lavelle and Sokol, 2020). It can exacerbate colitis by disrupting the epithelial barrier and inducing aberrant inflammation (Liu et al., 2020). Meanwhile, oral dysbiosis in patients with periodontitis can directly modulate the pathogenesis of IBD through the recruitment of the oral-gut axis (She et al., 2020). Specifically, *P. gingivalis*, a key pathogen of periodontitis, destroyed the gut barrier function by inducing the depression of tight junction proteins and causing dramatic alterations in the gut microbiome, including the enrichment of the Clostridiaceae family, thus promoting the gut and systemic inflammation (Kato et al., 2018; Kobayashi et al., 2020).

The imbalance of intestinal microecology due to *H. pylori* infection may also be associated with a series of other systemic diseases. Numerous retrospective cohort studies indicated that there was a correlation between positive *H. pylori* serological test, gut flora disturbance, and the incidence of Alzheimer’s disease (Baj et al., 2021). The gut microbiota creates a natural protective barrier, and secrets numerous neurotransmitters and neuromodulators, such as serotonin, γ-aminobutyric acid, dopamine, or SCFA including acetate, propionate, and butyrate to defend against microorganisms and endotoxin translocation (Paone and Cani, 2020), however, the *H. pylori* infection could remodel the gut microbiota, which may affect the function of the nervous system by inducing the degeneration and loss of neurons (Baj et al., 2021). Other systematic diseases related to gut microbiota, such as inflammation, dyslipidemia, hyperglycemia, arteriosclerosis, and hypertension could also be affected by *H. pylori* infection through its impacts on the changes in gut microbiota (Beydoun et al., 2018). However, its specific mechanism of action remains to be explored.

Probiotics appear to be an effective way to prevent and treat related diseases by regulating the balance of microbiota. Probiotics are defined as living microbial species, including bacteria and yeast, with the capabilities of regulating the host immune functions or by preserving the balance of intestinal flora, promoting nutrient absorption and maintaining intestinal health (Chee et al., 2020; Da Silva et al., 2021). They can be used to treat gastrointestinal disorders, sometimes in combination with other drugs (Domingo, 2017), to improve the balance of gut microbiota, enhance the production of short-chain fatty acids, and interact with host cells such as immune, nerve, and endocrine cells in the gastrointestinal tract (Hori et al., 2020). Therefore, for *H. pylori*-infected patients, probiotics may be an effective alternative to the antibiotic eradication therapy of *H. pylori*. The mechanisms of probiotics acting on *H. pylori* are mainly due to the following ways: 1. Probiotics secreted various antibacterial substances such as lactic acid, acetic acid, and hydrogen peroxide to inhibit the growth of *H. pylori* (Kim et al., 2003); 2. Probiotics can adhere to the receptor through non-specific competition to inhibit the adhesion of *H. pylori* on the gastric epithelial cells (Johnson-Henry et al., 2004); 3. Probiotics can restore the secretion of gastric mucus, which was significantly reduced in the patients’ gastritis because of the gastric epithelium damage or proliferation (Mack et al., 2003); 4. Probiotics can interact with epithelial cells and promote the secretion of anti-inflammatory cytokines to reduce the host’s immune response (Gill, 2003). Therefore, probiotics can be used as new agents to regulate *H. pylori* infection and its effects on gut microbiome by providing effective prevention and control of gastrointestinal diseases.

## Oral–gut microbiota in gastric disease and cancer

The pathogenicity of *H. pylori* mainly depends on its flagella, helical structure, lipopolysaccharide, cytotoxin-related protein, vacuolar toxin, and other pathogenic factors (Yamaoka, 2010), and its infection is closely related to chronic gastritis, peptic ulcer, gastric mucosa-associated lymphoid tissue lymphoma, gastric cancer, and other diseases. *H. pylori* also showed synergistic effect on the oral-gut axis commensal microbiota (Wong et al., 2019). Bacteria in the oral cavity and gut may be associated with the gastric cancer and be served as a diagnostic biomarker for gastric cancer (Park et al., 2021). For example, oropharyngeal or intestinal commensals such as *Streptococcus*, *Bifidobacterium*, *Lactobacillus*, *Veilonella*, *Klebsiella*, *Escherichia*, *Pseudomonas*, *Neisseria*, *Staphylococcus*, and *Bacillus* were all related to the development of gastric cancer as patients with gastric cancer had higher bacterial counts of these species than patients with other gastric diseases (Chen et al., 2021a).

The oral and gut are anatomically belonged to the digestive tract and are well-linked physically and chemically (Park et al., 2021). Under healthy conditions, the microbiota of these two habitats is separated, but under pathological conditions, they may enhance their communications (Seedorf et al., 2014). Common oral flora, such as *Porphyromonas*, *Fusobacterium*, *Pseudomonas*, *Haemophilus*, and *Veillonella*, can be detected in the gut of the elderly and patients with low gastric acid (Odamaki et al., 2016; Iwauchi et al., 2019). The stomach is anatomically located between the oral cavity and the gut. The gastric diseases are also significantly related to the microbiome of the oral-gut axis (Olsen and Yamazaki, 2019). In the oral cavity of chronic gastritis caused by *H. pylori* infection, low levels of *F. nucleatum* and *P. gingivalis* were detected (Contaldo et al., 2021). In the oral cavity of gastric cancer patients, pro-inflammatory taxa such as *Corynebacterium* and *Streptococcus* were enriched, but *Haemophilus*, *Neisseria*, *Parvimonas*, *Peptostreptococcus*, *Porphyromonas*, and *Prevotella* were reduced, indicating that the salivary microbiota was involved in gastric cancer pathogenesis by inducing the accumulation of pro-inflammatory bacteria and the reduction of carcinogenic N-nitroso compounds as *Haemophilus* and *Neisseria* can reduce the nitrite (Huang et al., 2021). Antibiotics and proton pump inhibitors which are used to treat *H. pylori* infection can reduce the diversity of microorganisms in the stomach and reduce gastric acid secretion to increase the translocation of microorganisms from the oral-gut axis, such as more oral predominant flora can be found in the stomach (Khor et al., 2021). However, the specific mechanisms remain to be further explored.

## Conclusion

*H. pylori* infection has a great impact on the microbiome of the oral-gut axis and has played important roles in the maintenance of host health and the development of oral, gut, and systematic diseases (The Integrative HMP (iHmp) Research Network Consortium, 2019; Park et al., 2021). However, their interactions are dynamic due to the possible reasons: 1. the growth conditions are different between *in vivo* and *in vitro* experiments; 2. the body’s immune defense and microbial composition are diverse under different host health conditions; 3. Different strains from the same genus or species may show different pathogenicity. Therefore, the interaction between *H. pylori* and oral-gut axis microbiota under different conditions is important for the prediction, prevention, and treatment of diseases, but the evaluations of detailed mechanisms are still needed.

## Author contributions

XC: Investigation, Writing – Original Draft Preparation, Validation. NW: Investigation, Writing – Original Draft Preparation. JW: Investigation. BL: Investigation. LC: Conceptualization, Funding Acquisition, Project Administration, Supervision. BR: Conceptualization, Funding Acquisition, Project Administration, Supervision, Writing – Review & Editing. All authors contributed to the article and approved the submitted version.

## Funding

This research received funding from the National Natural Science Foundation of China, Grant numbers: 81870778, 81600858, 81870759, 81870754, Applied Basic Research Programs of Sichuan Province, Grant number 2020YJ0227, Sichuan Province Key R&D Program, Grant number 2021YFQ0064. The funders had no role in the study design, data collection, and analysis, decision to publish, or preparation of the manuscript.

## Conflict of interest

The authors declare that the research was conducted in the absence of any commercial or financial relationships that could be construed as a potential conflict of interest.

## Publisher’s note

All claims expressed in this article are solely those of the authors and do not necessarily represent those of their affiliated organizations, or those of the publisher, the editors and the reviewers. Any product that may be evaluated in this article, or claim that may be made by its manufacturer, is not guaranteed or endorsed by the publisher.
